# Design and Implementation of an Electrical Characterization System for Membrane Capacitive Deionization Units for the Water Treatment

**DOI:** 10.3390/membranes11100773

**Published:** 2021-10-11

**Authors:** Federico A. Leon, Alejandro Ramos-Martin, David Santana

**Affiliations:** Departamento de Ingeniería de Procesos (Process Engineering Department), Campus de Tafira, Universidad de las Palmas de Gran Canaria (University of Las Palmas de Gran Canaria), 35017 Las Palmas de Gran Canaria, Spain; alejandro.ramos@ulpgc.es (A.R.-M.); david.santana122@alu.ulpgc.es (D.S.)

**Keywords:** membranes, capacitive deionization, reverse osmosis, desalination

## Abstract

The desalination of seawater is one of the most established techniques in the world. In the middle of the 20th century this was achieved using water evaporation systems, later with reverse osmosis membranes and nowadays with the possibility of capacitive deionization membranes. Capacitive deionization and membrane capacitive deionization are an emerging technology that make it possible to obtain drinking water with an efficiency of 95%. This technology is in the development stage and consists of porous activated carbon electrodes, which have great potential for saving energy in the water desalination process and can be used for desalination using an innovative technology called capacitive deionization (CDI), or membrane capacitive deionization (MCDI) if an anion and cation membrane exchange is used. In this paper is proposed and designed a characterization system prototype for CDI and MCDI that can operate with constant current charging and discharging (galvanostatic method). Adequate precision has been achieved, as can be seen in the results obtained. These results were obtained from the performance of typical characterization tests with electrochemical double layer capacitors (EDLC), since they are electrochemical devices that behave similarly to MCDI, from the point of view of the electrical variables of the processes that take place in MCDI. A philosophy of using free software with open-source code has been followed, with software such as the Arduino and Processing programming editors (IDE), as well as the Arduino Nano board (ATmega328), the analogical-digital converter (ADC1115) and the digital-analogical converter (MCP4725). Moreover, a low-cost system has been developed. A robust and versatile system has been designed for water treatment, and a flexible system has been obtained for the specifications established, as it is shown in the results section.

## 1. Introduction

The desalination of seawater is one of the most established techniques in the world. In the middle of the 20th century, this was achieved using water evaporation systems and later with reverse osmosis membranes. However, the resulting high energy consumption, generally through fossil fuels, has been an important issue to solve [1,2,3,4].

Modern times have introduced the possibility to transition from the use of reverse osmosis to capacitive deionization (CDI), or membrane capacitive deionization when an anion and cation membrane exchange is used, which is a novel system still under development that, in addition to removing salt from the water, allows for the storage of energy, similar to other electrochemical devices (batteries, supercapacitors, etc.) [5,6,7,8].

Currently, some media outlets are claiming that the current hydrological year is the driest hydrological year of the decade. In the last 12 months, the Spanish water reserve has decreased by a volume of water equivalent to that of the Ebro Basin (7511 hm^3^ of capacity).

These phenomena are aggravated by the evident signs of climate change and, in countries such as Spain, by the advance of desertification processes in large areas, causing water to become so much of an essential resource that it has become as precious a commodity as oil [4].

Knowing that 97% of the planet’s water is salty, the desalination of brackish or marine water (or seawater desalination) is one of the possible alternatives to mitigate the problem. However, current desalination technologies, such as reverse osmosis and distillation present a serious problem, which is the high energy consumption required to produce drinkable water, as well as the problem of boron removal.

Capacitive deionization (CDI) is an emerging technology that makes it possible to obtain drinking water with an efficiency of 95% [5,6,7,8,9,10]. This technology is in the development stage and consists of porous activated carbon electrodes, which have great potential for saving energy in the water desalination process and can be used for desalination using an innovative technology called capacitive deionization (CDI) and membrane capacitive deionization (MCDI). In CDI and MCDI, salt ions are removed from brackish water by applying a voltage difference between two porous electrodes in which the ions are temporarily immobilized, and dissolved salts are removed from different types of water [6,7,8,9].

The design and implementation of a characterization system for capacitive deionization units for water treatment was performed using types of water ranging from supply water to water from industrial processes. Capacitive deionization has emerged over the years as a robust, energy-efficient solution for water treatment [10,11,12,13,14,15,16,17,18]. It is an effective technology for the desalination of water with low to moderate salt content [5].

The optimal operating regime is obtained for water with a salt concentration of slightly less than 10 g/L [18]. Since the salt ions are minority compounds in the water, they can be removed from the mixture with this technology. In contrast, other methods extract the water phase from the salt solution. This technology also allows for the possibility of integrating renewable energy sources and energy storage [6,7,8,9,10].

The main objective of this study was the design and implementation of an electrical characterization system for capacitive deionization units in water treatment. The intention was also to make use of this system to obtain results that will make it possible to analyze the impact of this technique on different CDI and MCDI elements. The electrical characterization system of CDI and MCDI has been implemented before [11,12,13]. In more detail, the objectives to be achieved are the following:

To study a control system with a microcontroller (ATmega328 MICROCHIP) that will allow interaction, control, conditioning of different tests and data collection with the medium.

For future implementations, to study the variation of the concentration as a result of the influence of injecting energy into the system, being influenced by the pH of the solution, temperature, conductivity, etc.

Analyze by numerical methods the variables involved in the system,

Develop a system capable of testing different situations that verify the tests described in similar works of the field.

Establish a laboratory-scale electrochemical evaluation methodology for the electrical characterization of CDI and MCDI.

In addition to the tests, the results obtained will be used to determine improvements in active materials (activated carbon, carbon fibers, carbon aerogels, etc.) and the current collectors that make up the electrodes.

The set of results generated will serve as didactic material to illustrate the influence of the effects studied. This material will be useful for future students and teachers of degrees and masters programs of different universities thanks to the research and improvements on the CDI and MCDI units.

## 2. Materials and Methods

**Figure 1:**
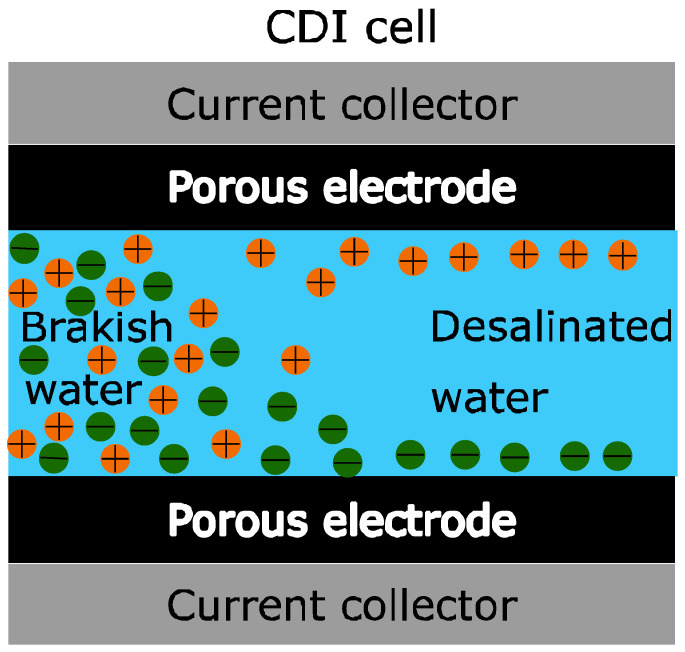
Capacitive deionization cell (CDI).

Compared to the classical working modes of the 20th century, there have been several innovations and new technologies, such as ion exchange in the membrane (see Figure 1) [14,15,16,17,18], and operational optimization in modes such as “stop-flow” during ion exchange [19], salt ratio upon voltage reversal [16], current constant during operation [19], energy recovered for desalination in relation to cycling [20,21], flow through electrostatic where water is directly faced through the electrostatic [22,23] and flow electrodes based on suspended carbon [24].

Regarding the historical evolution of CDI and MCDI, a stage was defined before 1995, when aerogel carbon was developed for CCD. The peons who worked with the desalination concept called it “electrochemical water demineralization” and its originators were Blair, Murphy and their colleagues, in the 1960s [25,26,27,28]. The basic standardized methods for supercapacitors have been used to study the behavior of CDI and MCDI [29,30,31,32,33]. These methods are constant current charging and discharging, constant power cycling, cyclic voltammetry and electrochemical impedance spectrometry. These procedures, and the self-charging and self-discharging phenomena, will be classified into three typologies: frequency, dynamic and energy characterization.

### 2.1. Frequency Characterization

The electrochemical impedance spectroscopy (EIS) method is used to characterize the electrochemical behavior of energy storage devices.

This method has gained popularity in the last decade, especially in the determination of the capacity in fuel cells and batteries, due to the development of the electric car [34,35].

The purpose of the EIS is to study the system response to the application of a small amplitude periodic alternating current (AC) signal. These measurements are carried out at different frequencies, which is why it is in the frequency characterization group. However, this technique is very sensitive, and it is essential to perform it as accurately as possible. Therefore, it is necessary to use several characterization techniques, because there is no single technique that gives all the expected results. Complementary techniques are therefore used.

On the other hand, there are limitations at high frequencies with CDI, due to the low impedance that the CDI cell can present. One of the factors conditioning the impedance of CDI is dissolution, while others are caused by coupling effects between the power supply and the media system cables [36]. These adversities are limited to low frequencies. In practice, twisted cables are used for the measurement cells to reduce these types of errors.

### 2.2. Dynamic Characterization

The constant current charge/discharge method is the simplest method to apply with direct current (DC). It analyzes the response of CDI when a constant charge/discharge current is applied to it. Despite being a basic method, it is widely used in the characterization of capacitors. Its application is documented with standardizations and will be the main object of the characterization of CDI in this work, being the main endorsement of the designed device [37,38,39,40].

The self-charging or self-discharging of CDI has its origin in two main causes. First, when CDI is charged or discharged, there is a diffusion of ions from the solution to the activated carbon electrode, or vice versa. The diffusion immediately after charging or discharging causes a self-discharge or self-charge, respectively. Secondly, these phenomena exist due to leakage current. Self-discharge by diffusion occurs predominantly in the initial stage. However, self-discharge by leakage is significant after self-discharge begins [41]. Self-discharge originates during dynamic operation, e.g., the dynamic operating cycle of electric vehicles [42], whose genome is through the equivalent impedance method, whose phase element is constant.

On the other hand, we highlight cyclic voltammetry (C.V.), which has achieved the distinction of a procedure used to evaluate the performance of energy storage systems, as well as to determine the life cycle, the effects of internal resistance and the dissipation losses [43]. The method consists of applying a linear voltage ramp and storing the current response in the capacitors. This current is directly associated with the capacity of the component achieving a versatile procedure [44,45,46,47,48,49].

Constant power cycling is known as “cycle life testing” (C.L.T.); it is a methodology for calculating the electrochemical device parameter based on periodic charging and discharging, separated by rest time intervals [50,51,52,53].

The procedure consists of charging the device at a given power until a voltage is achieved [54,55,56,57]. Once this point is reached, the polarity of the current is reversed by applying a given value, and the current is resumed by reversing the current, so that the charging cycle is continued up to a higher voltage [45,46].

**Figure 2:**
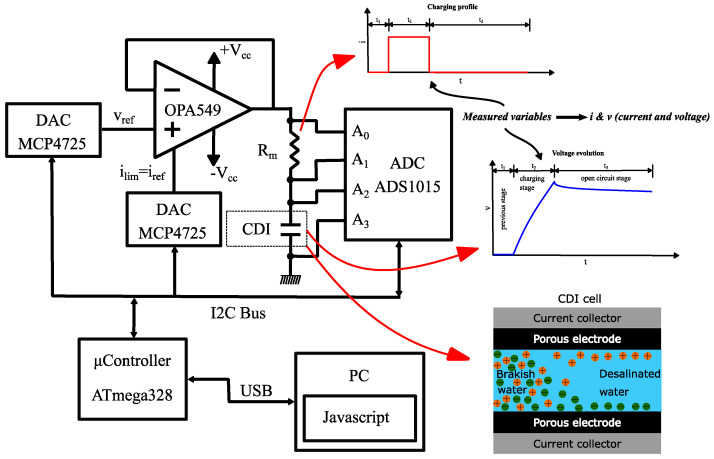
Scheme of the characterization system prototype for capacitive deionization (CSP-CDI).

This is a research study in the industrial field and several milestones have been previously established, which are as follows:

1. A device capable of characterizing capacitive deionization (CDI) and membrane capacitive deionization (MCDI) for different tests, shown in Figure 2, in such a way that the main characteristics of capacitive deionization can be obtained, such as equivalent series resistance, capacitance, etc.
2. A system capable of collecting the test parameters and configuring it through a graphical user interface (GUI).
3. A procedure capable of characterizing the CDI and MCDI for stationary flows.
4. The results obtained will be studied in future research lines for CDI and MCDI technology.

### 2.3. General Description of the System

**Figure 3:**
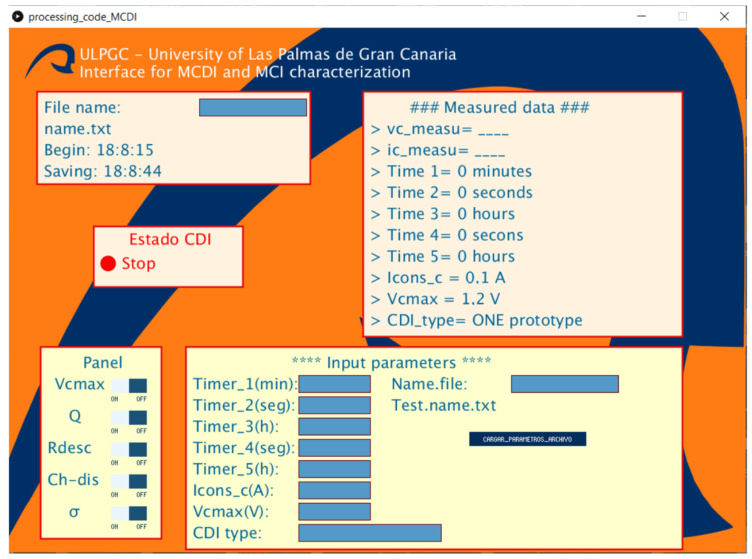
Processing PC control interface and data logger.

Figure 2 shows the scheme of the characterization system prototype for capacitive deionization (CSP-CDI), and the interrelation of components. These components that make up this diagram define the fundamental structure of the system. It is briefly explained below:

1. Control and data acquisition system (microcontroller ATmega328): this part of the system can store data and control the different parameters of the CSP-CDI. This part of the system can analyze the data in real time to actuate the power system. The microcontroller ATmega328 model (Atmel, Beijing, China) is implemented in the platform Arduino Nano (Arduino, Beijing, China). This microcontroller is responsible for directly controlling the galvanostatic charging process, so as to capture the data and send them to a PC for saving them (Appendix A shows the Arduino source code).
2. Power system (OPA549): A power operational amplifier OPA549 (Texas Instruments). This operational amplifier can provide a nominal current of 8A and also has a special input (ilim) for limiting the output current of the amplifier. This is an important feature for implementing galvanostatic charge or discharge (by controlling the current). This operational amplifier is responsible for sourcing the system, in order to charge and discharge the CDI cell or other similar electrochemical devices (batteries, electrochemical double layer capacitors, etc.).
3. ADS1015: An analogical-digital conversion stage based on the precision converter ADS1015 (Texas Instruments, Shanghai, China). This stage has two differential analog inputs for measuring: the charging or discharging current and the voltage of CDI cell. Current i(t) is measured from the voltage of a resistor Rm = 0.1 Ω, which is arranged between the output of the operational amplifier and CDI cell and the CDI cell voltage v(t) directly. The circuit ADS1015 has a 12-Bit resolution, which sets a resolution of 3 mV for the voltage measuring and 30 mA for the current measuring.
4. MCP4725: Two digital-analogical conversion stages based on MCP4725 (MICROCHIP, Beijing, China) with a resolution of 12 Bit. These stages are used to control the charging/discharging processes of EDLC, for setting the voltage and current intensity references.
5. A PC: that is responsible for controlling and configuring the microcontroller for conducting the tests and storage of data obtained from the measurements. This is achieved through a script made in JavaScript language under Processing IDE (Processing Foundation). For the different tests, data were sent to the PC from the microcontroller (ATmega328), where they were saved by means of the use of Processing tool. At the Appendix B is showed the Processing source code, and Figure 3 displays the control interface achieved with Processing, for being used at the PC.
6. CDI cell: this is the object of study of this paper. Its correct operation is essential to characterize it. Its operating range has been designed to satisfy the needs required in the programmed tests, which have carried on with different EDLCs (electrochemical double layer capacitors) for commissioning and evaluation.

## 3. Results

The target prototype developed for characterization testing is capable of charging and discharging at constant voltage and current (galvanostatic mode). In addition, the power supply provides the load with a constant current at 95% efficiency and establishes the duration for which the nominal voltage is maintained. The device is capable of measuring current and voltage with a maximum error tolerance of ±1%. Moreover, voltmeter measurements have a resolution of 0.125 mV for voltage measurement. The input impedance of these voltmeters must be sufficiently high for measurement errors to be negligible. To obtain the data, an analog-digital converter (ADC1115) was used, which has a series of filters at the input and output of the converter, resulting in precise, noise-free data.

The designed device must be able to perform constant voltage and current charging and discharging, as well as simultaneous voltage and current measurements at the terminals of the CDI cell.

The power supply (through the OPA549) must be able to provide the constant load current to the CDI cell with 95% efficiency and, in addition, set the duration of voltage maintenance.

The developed device must satisfy the requirement of an error tolerance of ± 0.01% for voltage measurement and an error tolerance of ±0.1% for current measurement.

### 3.1. Proposed Test Results

This section provides the details of the tests carried out and the results obtained for the characterization of the CCD system.

#### 3.1.1. Calibration of the Equipment

To provide veracity and reliability to the results obtained during the tests, the CSP-CDI was first subjected to a series of calibration tests. The procedure that followed consisted in the fact that, knowing an input setpoint, the system returned a series of expected results since the output data were known, since a 1 Ω resistor was used instead of a CDI cell.

##### Calibration of the Digital-to-Analog Converter DAC (MCP4725)

The first test of the CSP-CDI was the calibration of the equipment by setting an input setpoint current to measure the voltage at the terminals of the 1 Ω resistor (see the results in Table 1). With this experience, the calibration line of the equipment is determined, obtaining the expression of the digital-analogical converter’s transformation of accounts.

**Figure 4:**
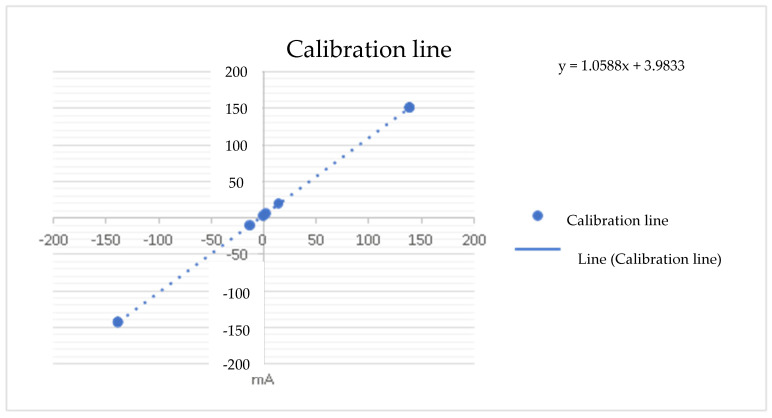
DAC calibration with the load resistance.

As can be seen in the previous Figure 4, the slope of the straight line corresponds to the resistance of 1 Ω, and it can also be seen that the data dispersion is null, so the sample taken refers to the population and indicates that the CSP-CDI is characterized with the Equation (1):

y = 1.0588 X + 3.9833(1)

where:

X is the rated setpoint current in mA (milli-Amperes).

Y is the current at the load mA (milli-Amperes).

Finally, knowing the current, the voltage is trivial because the resistance is 1 Ω.

##### Calibration of the Analogical-Digital Converter (ADS1115)

**Figure 5:**
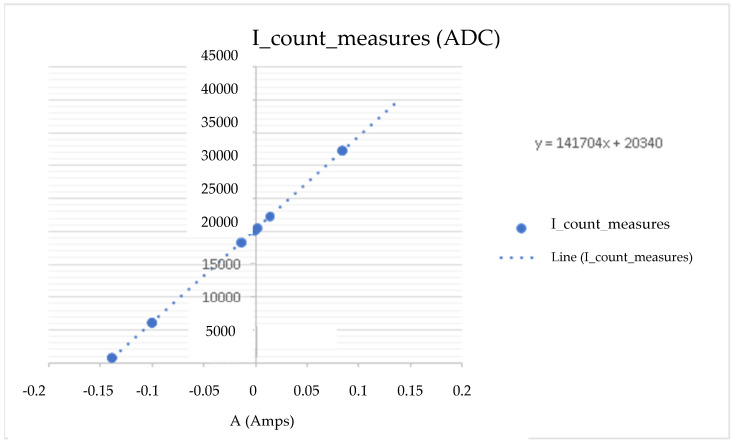
“I” counts measured at the ADC.

**Figure 6:**
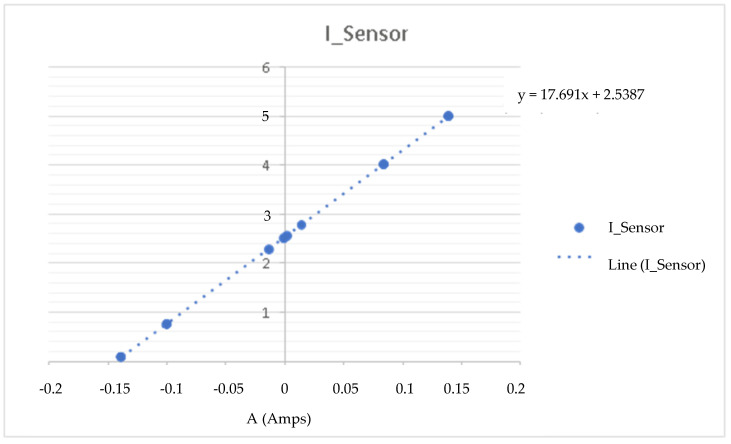
Intensity at the sensor.

**Figure 7:**
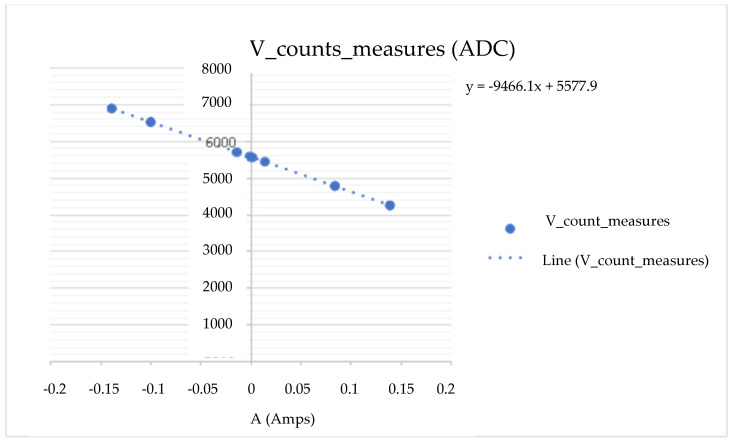
“V” counts measured on the ADC.

**Figure 8:**
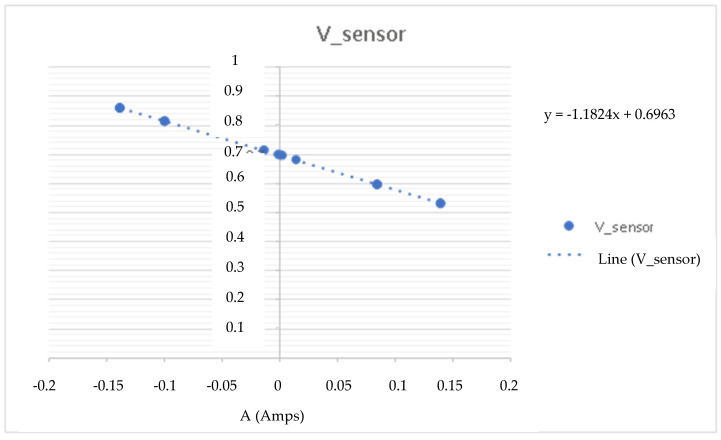
Voltage at the sensor.

This experiment is based on connecting a 1 Ω (Ohms) resistor to the load and establishing an input setpoint current to measure the voltage in the measuring cell. With this, we know the current and current of the CSP-CDI (see Figure 5, Figure 6, Figure 7 and Figure 8). With this experience, the calibration line of the equipment is determined, obtaining the expression of the transformation of accounts of the analogical-digital converter (Table 2).

As can be seen in the previous figures, the data dispersion is null, so the sample taken refers to the population and indicates that the CSP-CDI is characterized, with Equation (2) being “I” of the measured ADC counts, while Equation (3) is the sensor current, Equation (4) is “V” of the measured ADC counts, and Equation (5) is the sensor voltage, as follows:

y = 141704 X + 20340(2)

where:

X is the rated set point current in A (Amps).

Y are the counts corresponding to the current of the ADS1115.

y = 17.691 X + 2.5387(3)

where:

X is the rated setpoint current in A (Amperes).

Y is the measured current in V (Volts) at the sensor.

y = −9466.1 X + 5577.9(4)

where:

X is the rated setpoint current in A (Amperes).

Y are the counts corresponding to the voltage of the ADS1115.

y = −1.1824 X + 0.6963(5)

where:

X is the rated setpoint current in A (Amperes).

Y is the measured voltage in V (Volts) at the sensor.

#### 3.1.2. Charging and Self-Discharge Tests at an Input Set Point

This section specifies the tests to which the equipment is subjected for its validation, where the degree of response from the equipment to an input setpoint will be determined, characterizing its behavior through the supercapacitors and the standardized tests.

Three samples were taken in each test to avoid uncertainty and mean error. Furthermore, the data obtained in the tests are representative of the CSP-CDI.

The first characterization test performed was the charging and self-discharging of a 1 F capacity supercapacitor with a setpoint voltage of 2 V and a setpoint current of 0.1 A. The data generated were stored in a database. The data generated have been saved in a plain text file with “.txt” extension for further processing.

**Figure 9:**
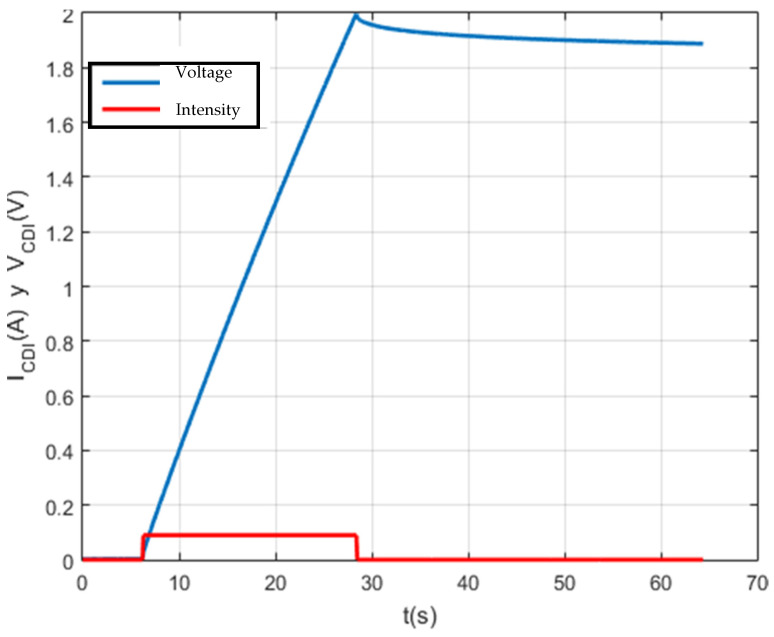
Charging and self-discharge test at Vc_max = 2 V and Ic = 0.1 A.

A script was created with Matlab software for data management on the screen. In this way, a detailed analysis of each test carried out is carried out. The result of this first test can be seen in Figure 9.

**Figure 10:**
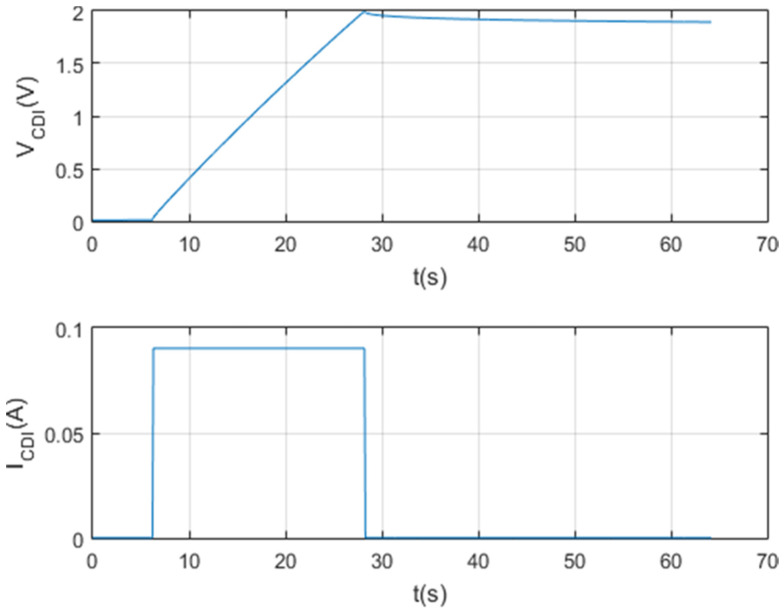
Charge and self-discharge test (voltage–current): Vc_max = 2 V and Ic = 0.1 A.

**Figure 11:**
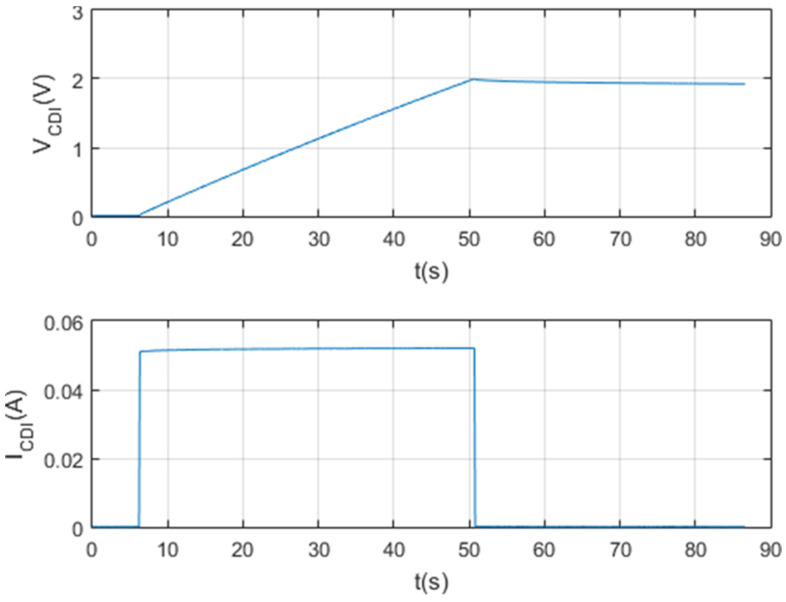
Load and self-discharge test at Vc_max = 2 V and Ic = 0.05 A.

**Figure 12:**
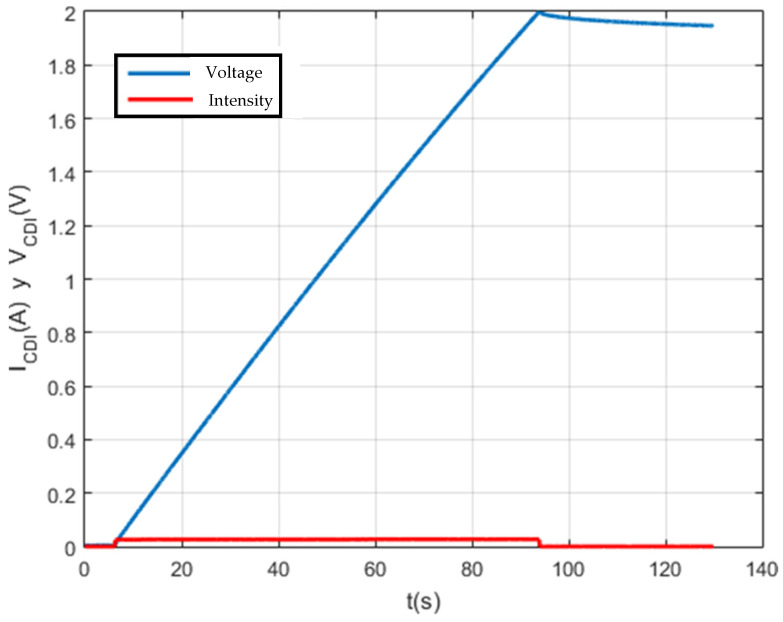
Charge and self-discharge test at Vc max = 2 V and Ic = 0.025 A.

**Figure 13:**
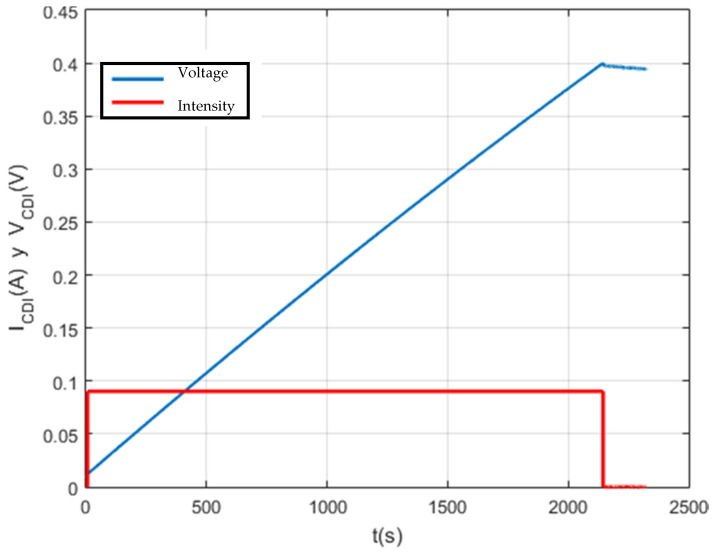
Test with a 650 F supercapacitor.

Figure 10, Figure 11, Figure 12 and Figure 13 show that the test was carried out satisfactorily. Although, there is a small variation of 0.01 A in the current. This was due to an equipment calibration problem, hence the importance of having the CSP-CDI properly calibrated.

The following tests were at the same maximum set point voltage, but at the set point current of 0.05 A and 0.025 A, respectively. The following Figure 12 and Figure 13 show the results of the tests per screen.

On the other hand, the system was validated with another supercapacitor, with a capacity of 650 F at another maximum voltage and setpoint current. The test results can be seen in the following figure.

**Figure 14:**
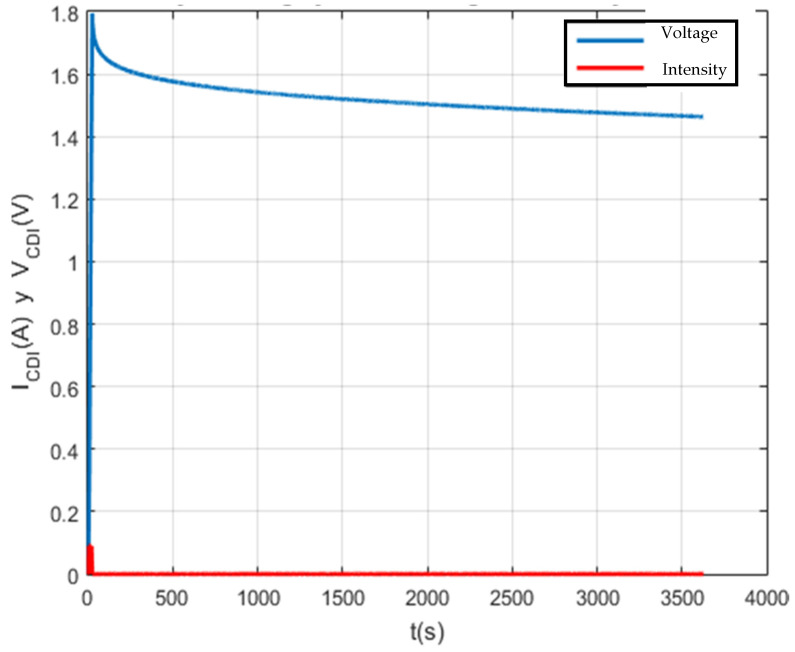
Self-discharge test to characterize a 1 F capacitor.

Firstly, as can be seen in Figure 14, the test was carried out at the set voltage of 0.4 V and set current of 0.1 A. This test lasted 1 h. It should be noted that the results are as expected for a supercapacitor with these characteristics, the reason being that the setpoint current is very small and the capacity of the supercapacitor is too large.

On the other hand, quality results have been obtained without the adverse effects of noise. This was possible because the design of the CSP-CDI foresaw the need to have a noise-free reading of the data when characterizing. For this reason, the use of the analogical-to-digital converter (ADS1115) is fundamental to the system. This converter has a series of input and output filters that result in accurate and noise-free data.

Finally, the self-discharge of CDI for 1 F, 100 F and 150 F supercapacitors has been analyzed and characterized (Figure 11, Figure 12, Figure 13 and Figure 14). The results shown are satisfactory and, with this, the characterization system is again validated.

**Figure 15:**
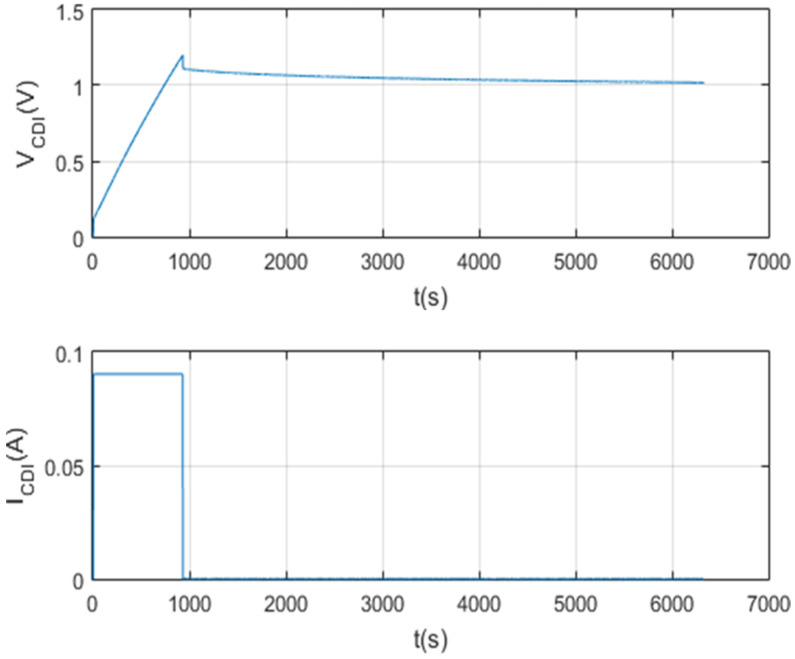
Self-discharge analysis characterizing a 100 F capacitor.

**Figure 16:**
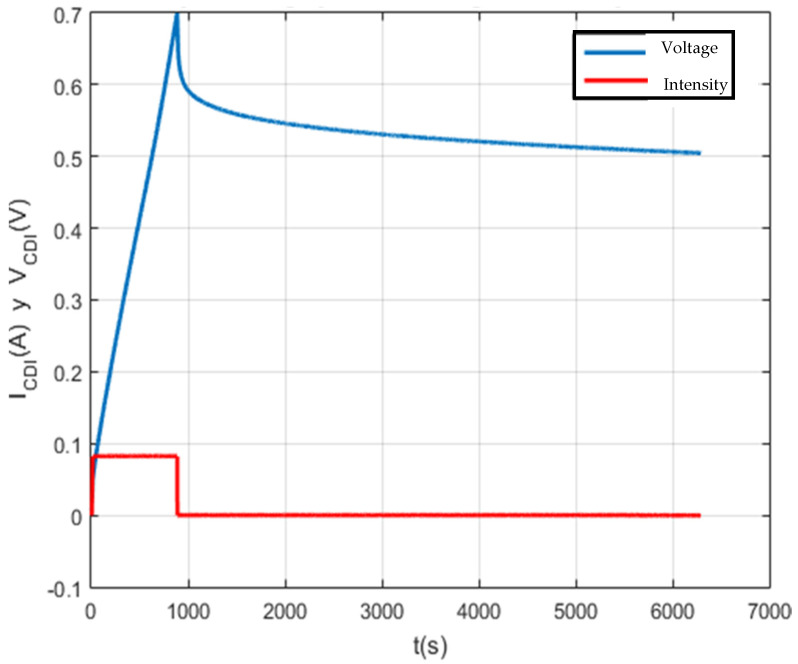
Charge and self-discharge test to characterize a 150 F capacitor.

**Figure 17:**
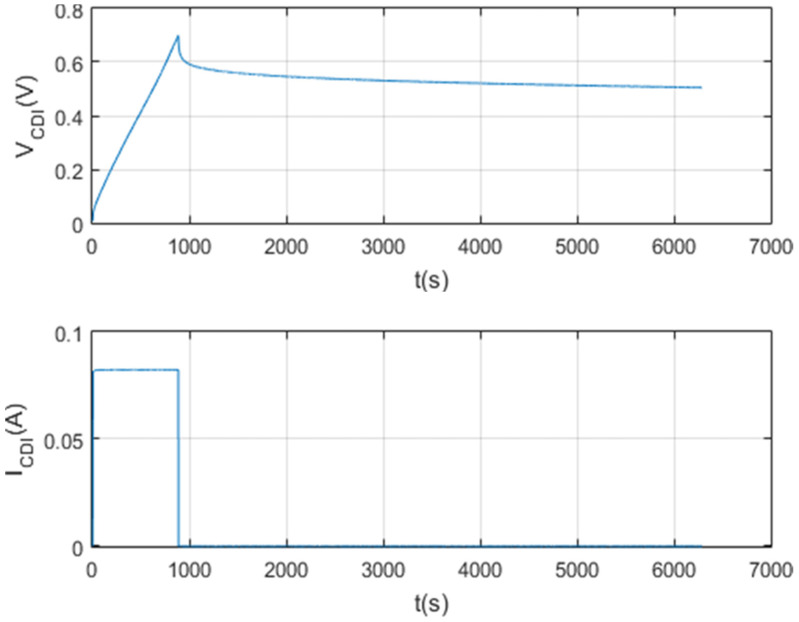
Graphs with details of the charge and self-discharge test, characterizing it on a 150 F capacitor.

As can be seen in Figure 15 and Figure 16, there are anomalies at the beginning of the charge and self-discharge. It has been deduced from the results obtained that an equivalent series resistance coexists due to the poor connection of the supercapacitor with the system terminals, resulting in a series resistance of 56 Ω. This can be seen in more detail in Figure 17.

#### 3.1.3. CDI Charging and Discharging Test

In this section, the system will be validated based on the standardized tests for supercapacitors. For the first charge and discharge characterization test of the CSP-CDI, the initial data have been programmed via the interface created with processing. The following table (Table 3) shows the data given to the interface.

**Figure 18:**
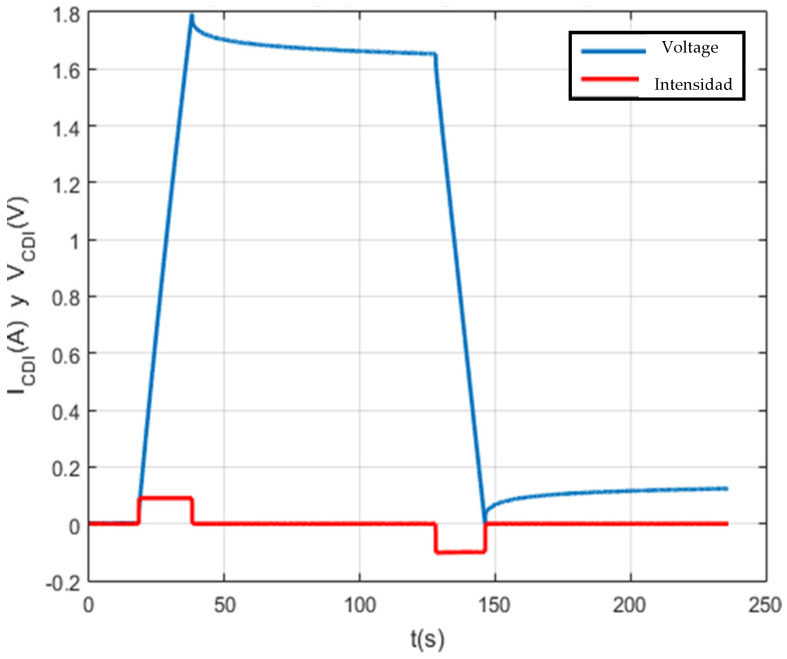
Charge and discharge test at Vcmax = 1.8 V and Icon = 0.1 A.

Once the data have been entered on the screen, the test is started. Once the test has been completed and the data obtained have been managed, the result is shown on the screen (Figure 18).

**Figure 19:**
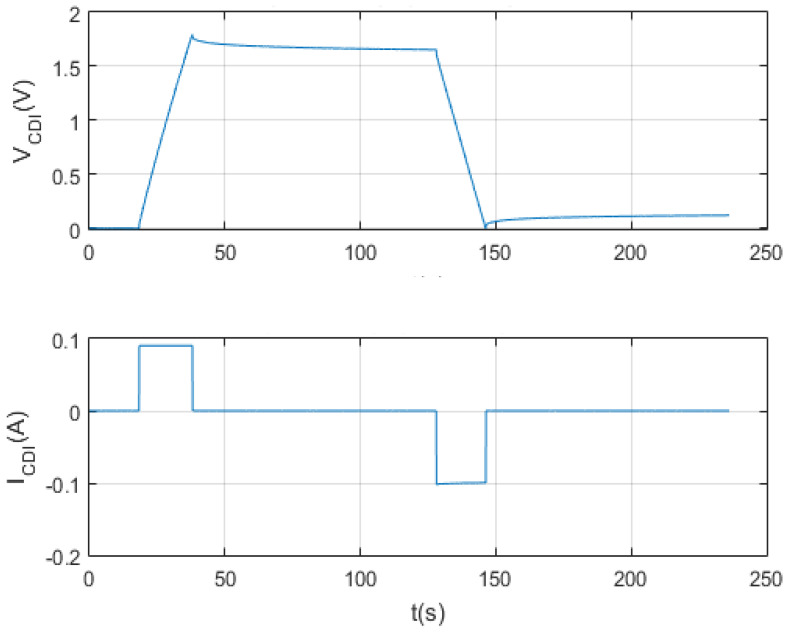
Detailed graphs for the loading and unloading test at Vcmax = 1.8 V and Icon = 0.1 A.

Firstly, the test is satisfactory. Figure 19 shows each of the times that make up the experiment, which are: time one, time two, time three, time four and time five.

Secondly, the test responds to the initially programmed times and the input setpoints (Vcmax and Icons).

Finally, the cleanliness of the data obtained should be highlighted, as the noise phenomenon in the result is negligible. This has been possible with the analogical-digital converter (ADS1115), which inhibits the noise generated by the instrumentation elements of the system.

On the other hand, another test has been carried out with different initial conditions to those described above, to validate the CSP-CDI system. The data entered by the interface are shown in Table 4.

**Figure 20:**
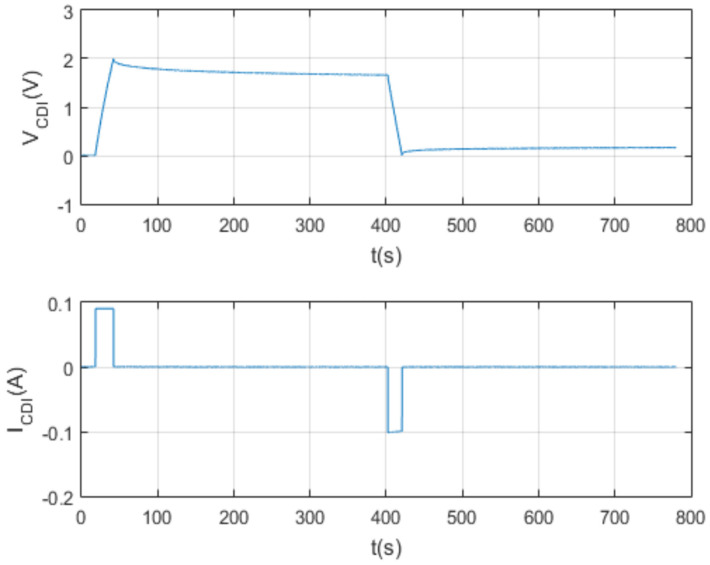
Charge and discharge test for the 1 F supercapacitor with other input setpoints.

The results per screen can be seen in Figure 20.

In Figure 20, it can be seen in time interval five that the capacitor self-charges once this period starts. The cause is the inertia of the capacitor in maintaining an equilibrium state, even though the attack current in the CSP-CDI system is 0A.

Finally, a new charge/discharge test is carried out with a supercapacitor of capacity 1 F (Farad), with different initial conditions to those previously described. The data initially programmed by the interface are shown in the following Table 5.

**Figure 21:**
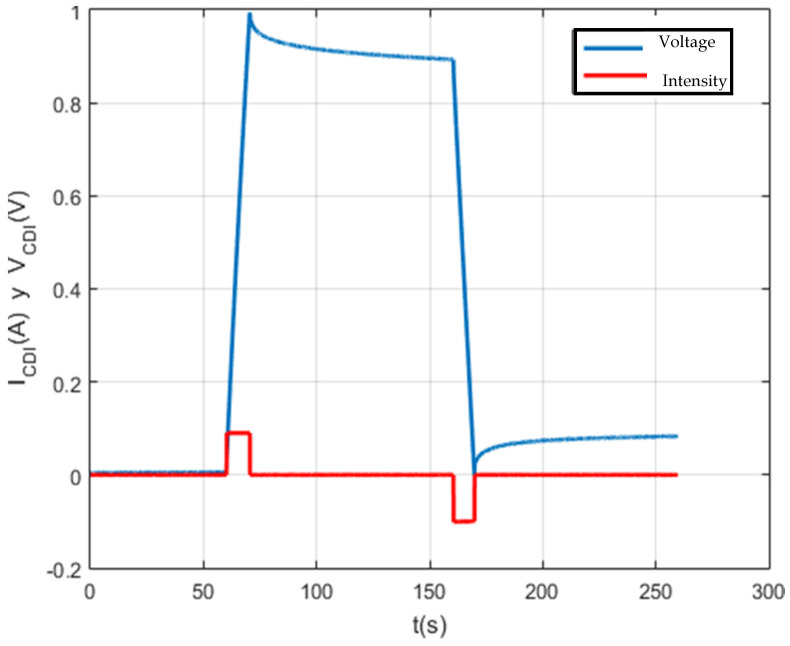
Charging and discharging test characterizing a 1 F supercapacitor.

**Figure 22:**
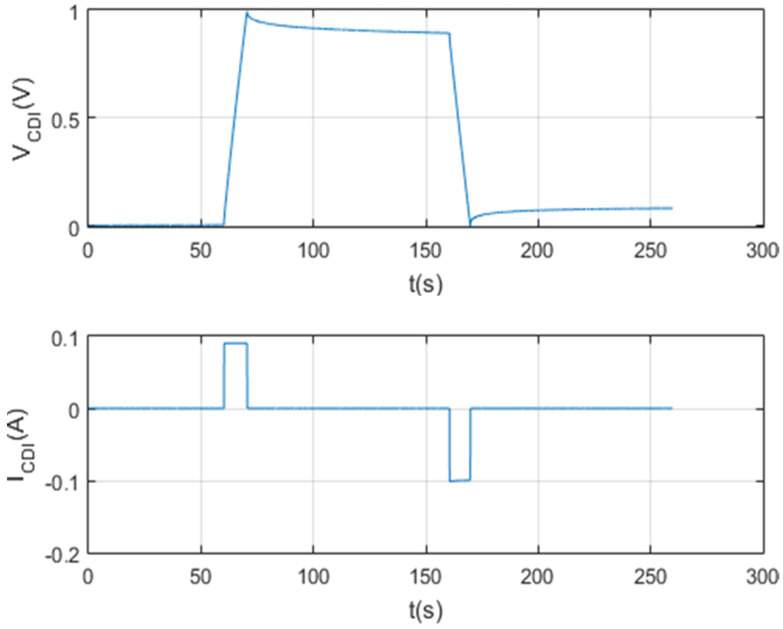
Charge and discharge test at Vcmax = 1 V and Icon = 0.1 A.

The following Figure 21 and Figure 22 show the results of the test per screen.

## 4. Conclusions

The conclusions obtained in the development of this study are as follows:

1. A prototype has been designed that can operate with constant current charging and discharging.
2. Adequate precision has been achieved, as can be seen in the results obtained.
3. A philosophy of using free software with open-source code has been followed, such as the microcontroller ATmega328 (Arduino platform) and Processing programming editors, as well as the analogical-digital converter (ADS1015) and the digital-analogical converter (MCP4725).
4. A low-cost system has been developed.
5. A robust and versatile system has been designed for water treatment.
6. A flexible system has been obtained for the specifications established.

## Figures and Tables

**Table 1:** Calibration of the system in the digital-analog converter (MCP4725).

Ic (mA)	Measures (mA)	
−138.890	−142.1	Test 1
−13.889	−10.6	
−1.3889	2.1	
0	3.9	
1.3889	5.6	
13.889	18.7	
138.890	151.5	
−138.890	−143.1	Test 2
−13.889	−10.5	
−1.3889	2.4	
0	3.6	
1.3889	5.3	
13.889	18.6	
138.890	151.4	
−138.890	−142.9	Test 3
−13.889	−10.5	
−1.3889	2.3	
0	3.7	
1.3889	5.4	
13.889	18.6	
138.890	151.4	

**Table 2:** Calibration of the system in the analog-digital converter (ADS1115).

V_Sensor	I_Sensor	V_Count_Measures	I_Count_Measures	Sample
0.860	0.091	6892	735	Measure 1
0.816	0.757	6527	6060	
0.713	2.292	5710	18,365	
0.698	2.515	5591	20,152	
0.696	2.541	5578	20,354	
0.695	2.563	5565	20,535	
0.680	2.784	5450	22,302	
0.597	4.020	4785	32,260	
0.860	0.090	6889	730	Measure 2
0.814	0.760	6524	6090	
0.713	2.290	5710	18,346	
0.698	2.515	5592	20,142	
0.696	2.542	5578	20,358	
0.695	2.565	5565	20,542	
0.680	2.783	5450	22,290	
0.597	4.020	4785	32,261	
0.860	0.090	6887	730	Measure 3
0.814	0.761	6524	6100	
0.713	2.292	5710	18,362	
0.698	2.515	5592	20,145	
0.696	2.543	5577	20,366	
0.695	2.565	5567	20,546	
0.680	2.783	5450	22,290	
0.597	4.020	4786	32,260	

**Table 3:** Data initially programmed in the processing interface.

Time 1	0.3	Minutes
Time 2	45	Seconds
Time 3	0.25	Hours
Time 4	45	Seconds
Time 5	0.25	Hours
I_c_	01	A (Amperes)
Vcmax	1.8	V (Volts)
Capacitor capacity	1	F (Farads)

**Table 4:** Data programmed for the second loading and unloading test.

Time 1	0.3	Minutes
Time 2	45	Seconds
Time 3	0.1	Hours
Time 4	45	Seconds
Time 5	0.1	Hours
Ic	0.1	A (Amperes)
Vcmax	2	V (Volts)
Capacitor capacity	1	F (Farads)

**Table 5:** Data programmed to characterize the CSP-CDI system.

Time 1	1	Minutes
Time 2	45	Seconds
Time 3	0.025	Hours
Time 4	45	Seconds
Time 5	0.025	Hours
Ic	0.1	A (Amperes)
Vcmax	1	V (Volts)
Capacitor capacity	1	F (Farads)

## Appendix A. Microcontroller Source Code (ATmega328)

/****************************************************************************************************

CSP_MCDI ATmega328 source code

Title: Design and implementation of an electrical characterization system for membrane capacitive deionization units for water treatment.

Authors: Federico Leon, Alejandro Ramos-Martin and David Santana-Quintana.

****************************************************************************************************/

// V2-2021/07/07

#include <TimerOne.h>

#include <Wire.h>

#include <Adafruit_ADS1115.h>

#include <Adafruit_MCP4725.h>

// Configuracion nombres del sistema ADS y DAC

Adafruit_ADS1115 ads;

//Adafruit_MCP4725 dac;

Adafruit_MCP4725 dac1;

// Configuración de los reles para conseguir sistemas aislados

const int Rele1 = 2;//Rele de control 1.

const int Rele2 = 3;//Rele de control 2.

int16_t a;

int16_t b;

int c = 0; // Consigna para los ensayos

/************************************************************************************

* Inicialización de variables

************************************************************************************/

unsigned long inicio=0;//Variable de entrada que fuerza la puesta en marcha y parada del ensayo.

// 0 -> En espera o parado.

// 1 -> Cargar con consigna de tension maxima.

// 2 -> Cargar con consigna de carga electrica.

// 3 -> forzar descarga con resistencia externa.

// 4 -> carga y descarga.

// 5 -> conductimetro.

unsigned long tiempo_1=0;//Tiempo de precarga.

unsigned long tiempo_2=0;//Tiempo de carga, cuando se establezca la referencia de carga eléctrica (inicio=2).

unsigned long tiempo_3=0;//Tiempo de estado de auto-descarga o tiempo de mantenimiento de la tensión

unsigned long tiempo_4=0;//Tiempo de estado de descarga.

unsigned long tiempo_5=0;//Tiempo de estado de auto-descarga.

uint16_t Vcmax=0;//Consigna de tensión máxima de carga, siempre se aplicará por seguridad.

int estado=1;//Variable de salida que indica en que modo de operación se encuentra la celda de CDI

// 0 -> En espera o stop, pero enviando datos.

// 1 -> En estado de precarga, durante tiempo_1.

// 2 -> En estado de carga, hasta que se complete el tiempo_2 o se alcance la tensión Vcmax para evitar la

// electrolisis

// 3 -> En estado de auto-descarga.

// 4 -> Finalizado el ensayo.

unsigned long control_tiempos=0;//Variable de control de tiempos en cada estado.

/* RESOLUCIONES del MCP4725

* Tensiones del Arduino:

* 0 V > 2.5 V ------>> Tensiones positivas 0–2048 bit para MCP4725

* 2.5 V > 5 V ------>> Tensiones negativas 2048–4095 bit para MCP4725

*/

//Expresión de cálculo; NOTA: Vx4=Gain*(Vx2-Vard)+Voffset; Gain=1;Voffset=0;

/*

* Vx4=(Vx2-Vard)

*/

/*********************************************************************

* Ventrada<>[−2.5;2.5]V // Valores de entrada al OPA548

********************************************************************/

uint16_t Vcuenta1=0;//Vopa ---->[X4=2.5 V]

uint16_t Vcuenta2=2048;//Vopa ---->[X4=0 V]

uint16_t Icons=2048;//Consigna establecida de corriente de carga-descarga.

// Iconns es un valor inicial que luego se refresca

int16_t results;

int16_t results1;

/************************************************************************************

* Configuración del puerto serial y del timerone

************************************************************************************/

void setup()

{

Serial.begin(9600);

pinMode(Rele1, OUTPUT);

pinMode(Rele2, OUTPUT);

digitalWrite(Rele1, HIGH);//HIGH--> NC en el relé 1

digitalWrite(Rele2, HIGH);//HIGH--> CDI descargada (NC en el relé 2)

Timer1.initialize(100000);// Configuración del timerONE

Timer1.attachInterrupt(intu);// Asignación de la subrutina a la interrupción del timerONE

dac1.begin(0 × 62); //Control de la corriente. Direccionamiento del conversor ADC

//dac.begin(0 × 63); //Control de la tensión. Direccionamiento del conversor ADC

ads.begin(); //Inicia el ADS1115.

ads.setGain(GAIN_ONE); // 1× gain +/− 4.096 V 1 bit = 2 mV 0.125 mV

// Establecimiento de la ganancia del ADC

/*

* Ventrada<>[2,5;−2,5] --> Rcerámica=18 ohm

* I_carga_max=2.5/18=138,9 mA

* Icarga maxima=138.9 mA --> {Icons=0}

* Icarga mínima=−138.9 mA -> {Icons=4095}

*/

/************************************************************************************

* Configuración de los DAC y los relés

************************************************************************************/

dac1.setVoltage(2048, false);//Inicializado a 0A.

digitalWrite(Rele1, HIGH);//Rele NC

digitalWrite(Rele2, HIGH);//Rele NC

/*

* El OPA esta como un seguidor de tensión y la CDI está a tierra

*/

}

//interructión a los 1 micros

void intu()

{

Serial.print(a);

Serial.print(“,”);

Serial.print(b);

Serial.print(“,”);

Serial.print(Icons);

Serial.print(“,”);

Serial.print(Vcmax);

Serial.print(“,”);

Serial.println(estado);

// Serial.print(“,”);

// Serial.println(inicio);

control_tiempos=control_tiempos+1;

}

/***********************************************************************************************

* Se realizan los ensayos

***********************************************************************************************/

void loop(void)

{

Timer1.detachInterrupt();//Se deshabilita la interrupcion de envio de datos

switch (inicio){

//Tiempo a 0 sg

case 0: //Estado de espera o parada.

digitalWrite(Rele1, HIGH);//Relé NC.

digitalWrite(Rele2, HIGH);//Relé NC.

results1 = ads.readADC_Differential_0_1();

results = ads.readADC_Differential_2_3();

a = results1;

b = results;

control_tiempos=0;

Timer1.attachInterrupt(intu); //Se habilita la interrupción de envio de datos.

break;

/*****************************************************************************************

* RESOLUCIONES del MCP4725

* Vard= 5 V − 2.5 V ------>> Tensiones positivas 4095–2048 bit para MCP4725

* Vard= 2.5 V − 0 V ------>> Tensiones negativas 2048–0 bit para MCP4725

******************************************************************************************/

case 1: //Carga con consigna de tensión máxima.

switch (estado){

case 1: //Forzar estado de precarga.

digitalWrite(Rele1, HIGH);//Rele abierto (NC)

digitalWrite(Rele2, HIGH);//Rele cabierto (NC)

//Tensión

//dac.setVoltage(4095, false);//Tensión en el OPA, X4= −2.5 V.

//Corriente

// Se le asigna un ‘0’ al OPA

dac1.setVoltage(2048, false);//Corriente en el OPA, X4= 0 A.

results1 = ads.readADC_Differential_0_1();

results = ads.readADC_Differential_2_3();

a = results1;

b = results;

if(control_tiempos>=tiempo_1){

estado=2;

control_tiempos=0;

}

Timer1.attachInterrupt(intu); //Se habilita la interrupción de envio de datos.

break;

case 2: //Forzar estado de carga sólo con límite de tensión.

// Estado de carga, hasta que se complete el tiempo_2 o se alcance la

// tensión máxima

digitalWrite(Rele1, LOW);//Relé abierto (NA).

digitalWrite(Rele2, LOW);//Relé abierto (NA).

//<<<[Corriente]>>>

dac1.setVoltage(Icons, false);//Falta asignar la corriente.

//<<<[Tensión]>>>

//dac.setVoltage(Vcuenta1, false);//Falta asignar la tensión.

results1 = ads.readADC_Differential_0_1();

results = ads.readADC_Differential_2_3();

a = results1;

b = results;

// Valor máximo de tensión 1,2 V o 1 V para evitar la electrolisis de la CDI

if (a > Vcmax) {

estado=3;//Estado de auto-descarga

control_tiempos=0;

digitalWrite(Rele1, HIGH);//Relé NC.

digitalWrite(Rele2, HIGH);//Relé NC.

// Corriente

dac1.setVoltage(2048, false);//corriente en el OPA, X4= OA. Se le

// asigna un ‘0’ al OPA

// Tensión

//dac.setVoltage(4095, false);//Tensión en el OPA, X4= −2,5 V.

}

Timer1.attachInterrupt(intu); //Se habilita la interrupción de envio de datos.

break;

case 3://Forzar estado de auto-descarga.

results1 = ads.readADC_Differential_0_1();

results = ads.readADC_Differential_2_3();

a = results1;

b = results;

if(control_tiempos>=tiempo_3){

//Tiempo_3--> Tiempo de estado de auto-descarga

estado=4;//Ensayo finalizado

inicio=0;

control_tiempos=0;

}

Timer1.attachInterrupt(intu); //Se habilita la interrupción de envio de datos.

break;

}

//Fin del switch (estado)

break;

case 2://Carga con consigna de carga eléctrica.

switch (estado){

case 1: //Forzar estado de precarga.

digitalWrite(Rele1, HIGH);//Rele NC.

digitalWrite(Rele2, HIGH);//Rele NC.

//<<<[Tensión]>>>

//dac.setVoltage(4095, false);//Aproximadamente en el OPA, X4= −2.5 V.

//<<<[Voltaje]>>>

dac1.setVoltage(2048, false);//Aproximadamente en el OPA, X4= 0 A.

results1 = ads.readADC_Differential_0_1();

results = ads.readADC_Differential_2_3();

a = results1;

b = results;

if(control_tiempos>=tiempo_1){

estado=2;

control_tiempos=0;

}

Timer1.attachInterrupt(intu); //Se habilita la interrupción de envio de datos.

break;

case 2://Forzar estado de carga con limite de tiempo 2.

digitalWrite(Rele1, HIGH);//Relé NC.

digitalWrite(Rele2, HIGH);//Relé NC.

//<<<[Corriente]>>>

dac1.setVoltage(Icons, false);//dac.setVoltage(3000, false);//Falta asignar la corriente.

//<<<[Tensión]>>>

//dac.setVoltage(Vcuenta1, false);//dac.setVoltage(3000, false);//Falta asignar la tension.

results1 = ads.readADC_Differential_0_1();

results = ads.readADC_Differential_2_3();

a = results1;

b = results;

if (a > Vcmax) {

estado=3;// Estado de auto-descarga

control_tiempos=0;

digitalWrite(Rele1, LOW);//Relé NA.

digitalWrite(Rele2, LOW);//Relé NA.

//<<<[Corriente]>>>

dac1.setVoltage(2048, false);//Aproximadamente en el OPA, X4=OA.

//<<<[Tensión]>>>

//dac.setVoltage(4095, false);//Aproximadamente en el OPA, X4=−2.5 V.

}

if(control_tiempos>=tiempo_2){

estado=3;// Estado de auto-descarga

control_tiempos=0;

digitalWrite(Rele1, HIGH);//Relé NC.

digitalWrite(Rele2, HIGH);//Relé NC.

//<<<[Corriente]>>>

dac1.setVoltage(2048, false);//Aproximadamente en el OPA, X4=OA.

//<<[Tensión]>>>

//dac.setVoltage(4095, false);//Aproximadamente en el OPA, X4=−2.5 V.

}

Timer1.attachInterrupt(intu); //Se habilita la interrupción de envio de datos.

break;

case 3:// Forzar estado de auto-descarga.

// En este estado la posibilidad de emplear un interruptor para realizar dos tipos

// de ensayos.

results1 = ads.readADC_Differential_0_1();

results = ads.readADC_Differential_2_3();

a = results1;

b = results;

if(control_tiempos>=tiempo_3){

estado=4;// Ensayo terminado

inicio=0;

control_tiempos=0;

}

Timer1.attachInterrupt(intu); //Se habilita la interrupción de envio de datos.

break;

}//Fin switch (estado)

break;

case 3:// Se fuerza la descarga indefinida con resistencia de descarga.

digitalWrite(Rele1, HIGH);//Relé NC.

digitalWrite(Rele2, HIGH);//Relé NC.

//<<<[Corriente]>>>

dac1.setVoltage(2048, false);//Aproximadamente en el OPA, X4= OA.

//<<<[Tensión]>>>

//dac.setVoltage(4095, false);//Aproximadamente en el OPA, X4= −2.5 V.

results1 = ads.readADC_Differential_0_1();

results = ads.readADC_Differential_2_3();

a = results1;

b = results;

control_tiempos=0;

Timer1.attachInterrupt(intu); //Se habilita la interrupción de envio de datos.

break;

case 4:// Carga y descarga de la CDI

switch (estado){

case 1: //Forzar estado de precarga.

digitalWrite(Rele1, HIGH);//Rele abierto (NC)

digitalWrite(Rele2, HIGH);//Rele cabierto (NC)

//Tensión

//dac.setVoltage(4095, false);//Tensión en el OPA, X4= −2.5 V.

//Corriente

// Se le asigna un ‘0’ al OPA

dac1.setVoltage(2048, false);//Corriente en el OPA, X4= 0 A.

results1 = ads.readADC_Differential_0_1();

results = ads.readADC_Differential_2_3();

a = results1;

b = results;

if(control_tiempos>=tiempo_1){

estado=2;

control_tiempos=0;

}

Timer1.attachInterrupt(intu); //Se habilita la interrupción de envio de datos.

break;

case 2: //Forzar estado de carga sólo con límite de tensión.

// Estado de carga, hasta que se complete el tiempo_2 o se alcance la

// tensión máxima

digitalWrite(Rele1, LOW);//Relé abierto (NA).

digitalWrite(Rele2, LOW);//Relé abierto (NA).

//<<<[Corriente]>>>

dac1.setVoltage(Icons, false);//Falta asignar la corriente.

//<<<[Tensión]>>>

//dac.setVoltage(Vcuenta1, false);//Falta asignar la tensión.

results1 = ads.readADC_Differential_0_1();

results = ads.readADC_Differential_2_3();

a = results1;

b = results;

// Valor máximo de tensión 1,2 V o 1 V para evitar la electrolisis de la CDI

if (a > Vcmax) {

estado=3;//Estado de auto-descarga

control_tiempos=0;

digitalWrite(Rele1, HIGH);//Relé NC.

digitalWrite(Rele2, HIGH);//Relé NC.

// Corriente

dac1.setVoltage(2048, false);//corriente en el OPA, X4= OA. Se le

// asigna un ‘0’ al OPA

// Tensión

//dac.setVoltage(4095, false);//Tensión en el OPA, X4= −2.5 V.

}

Timer1.attachInterrupt(intu); //Se habilita la interrupción de envio de datos.

break;

case 3:

//<<<[Corriente]>>>

dac1.setVoltage(Icons, false);//Falta asignar la corriente.

//<<<[Tensión]>>>

//dac.setVoltage(Vcuenta1, false);//Falta asignar la tensión.

results1 = ads.readADC_Differential_0_1();

results = ads.readADC_Differential_2_3();

a = results1;

b = results;

if (){

}

break

case 4:

//<<<[Corriente]>>>

dac1.setVoltage(Icons, false);//Falta asignar la corriente.

//<<<[Tensión]>>>

//dac.setVoltage(Vcuenta1, false);//Falta asignar la tensión.

results1 = ads.readADC_Differential_0_1();

results = ads.readADC_Differential_2_3();

a = results1;

b = results;

// Valor máximo de tensión 1,2 V o 1 V para evitar la electrolisis de la CDI

if (a > Vcmax) {

estado=3;//Estado de auto-descarga

control_tiempos=0;

digitalWrite(Rele1, HIGH);//Relé NC.

digitalWrite(Rele2, HIGH);//Relé NC.

// Corriente

dac1.setVoltage(2048, false);//corriente en el OPA, X4= OA. Se le

// asigna un ‘0’ al OPA

// Tensión

//dac.setVoltage(4095, false);//Tensión en el OPA, X4= −2.5 V.

}

Timer1.attachInterrupt(intu); //Se habilita la interrupción de envio de datos.

break

case 5://Forzar estado de auto-descarga.

results1 = ads.readADC_Differential_0_1();

results = ads.readADC_Differential_2_3();

a = results1;

b = results;

if(control_tiempos>=tiempo_3){

//Tiempo_3--> Tiempo de estado de auto-descarga

estado=4;//Ensayo finalizado

inicio=0;

control_tiempos=0;

}

Timer1.attachInterrupt(intu); //Se habilita la interrupción de envio de datos.

break;

}

case 5:// conductimetro

// Aquí hay que implementar el código del conductimetro

//*************** Prueba *****************************

//#include <AD9833.h>

//

//const int fsync_pin= 6;

//const int sdata_pin = 7;

//const int sclk_pin = 8;

//

//static AD9833 DDS (sdata_pin, sclk_pin, fsync_pin);

//float F=1000;

//int PH=0, ch=0, s=0;

//void setup() {

//

//// pick a mode

//DDS.set_mode (s, ch); // values for s: 0=sine, 1=triangle, 2=square, ch = channel 0 or 1

//// To change settings:

//

//DDS.set_freq (F, ch); // F = output frequency in hertz, ch = channel 0 or 1

//DDS.set_phase (PH, ch); // PH = phase 0 degrees to 359.999, c = channel 0 or 1

//

//}

//

//void loop() {

// // put your main code here, to run repeatedly:

//

//}

break;//Se rompe el case

}//fin switch(inicio)

}//fin void loop()

/***********************************************************************************************

* Etapa del serial Event

***********************************************************************************************/

/*

* C ---> La consigna del ensayo{CASO -}

* Por ejemplo:

* Inicio --> 2

* Tiempo_1-> 50

* Tiempo_2-> 200

* Tiempo_3-> 50

** Tiempo_4-> 100

** Tiempo_5-> 100

* Icons --> 1500

* Vcmax --> 1000

*/

void serialEvent(){

////**1**//c 2,50,200,50,1500,1000 c 1,50,200,50,1500,1000 c 3,50,200,50,1500,1000

///***2***//c 2,50,200,50,100,100,1500,1000

Serial.println(“holaaaaa estoy aquiii David”);// Línea de prueba del SerialEvent

if (Serial.peek() == ‘c’) { //Verifica el caracter significativo para que pueda ser comandado

Timer1.detachInterrupt();//Se deshabilita la interrupción de envio de datos

Serial.read(); //Lee el puerto serial

inicio = Serial.parseInt();

tiempo_1 = Serial.parseInt();

tiempo_2 = Serial.parseInt();

tiempo_3 = Serial.parseInt();

tiempo_4 = Serial.parseInt();

tiempo_5 = Serial.parseInt();

Icons = Serial.parseInt();

Vcmax = Serial.parseInt();

estado=1;//Inicia el primer estado del ensayo.

control_tiempos=0;

Timer1.attachInterrupt(intu); //Se habilita la interrupción de envio de datos.

}

while (Serial.available() > 0){

Serial.read();

}

}

## Appendix B. Processing Source Code (ATmega328)

/****************************************************************************************************

CSP_MCDI GUI

Title: Design and implementation of an electrical characterization system for

membrane capacitive deionization units for water treatment.

Authors: Federico Leon, Alejandro Ramos-Martin and David Santana-Quintana.

****************************************************************************************************/

// V2-2021/07/07

import processing.serial.*;

import controlP5.*;

String nom_archivo=“name.txt”,para_nom_archivo=“name.txt”,estado_CDI=“Stop”;

String hora_inicio=“Begin: “+hour()+”:”+minute()+”:”+second();

String tiempo_1=“0”,tiempo_2=“0”,tiempo_3=“0”, tiempo_4=“0”, tiempo_5=“0”, tipo_CDI=“ONE”;

String I_c=“0,1”,V_c=“1,2”;

String tipo_ensayo=“0”;

PrintWriter archivo;//Archivo de datos ensayo.

PrintWriter fichero;//Parametros del ensayo.

ControlP5 cp5;

Serial myPort;

boolean serialInited;

int muestras_segundo=10;

int finalizado=0;

int xPos = 1;

int esquina_x=600;

int esquina_y=100;

int ancho=1200;

int alto=600;

int pocision_alto=0;

int guardar_datos=−1,inicio=0;

String dato_1=“____”,dato_2=“____”,dato_3=“____”,dato_4=“____”,dato_5=“____”,dato_6=“____”;

PShape bot;

// Configuración de los botones

int ancho_ventana=3020,alto_ventana=1500;

int x_r1=500,y_r1=90,dx_r1=450,dy_r1=345;

int mx_r1=10,my_r1=30,dy_t=30;

int x_r2=40,y_r2=90,dx_r2=385,dy_r2=130;

int mx_r2=10,my_r2=30;

int ancho_campo_texto=210;

int x_resto=40,y_resto=250,dy_resto=25;

int x_r3=40,y_r3=300,dx_r3=210,dy_r3=35;

int mx_r3=10,my_r3=30;

int ancho_boton=110,alto_boton=20,ancho_mitad_boton=55;

int x_r4=250,y_r4=450,dx_r4=700,dy_r4=285;

int mx_r4=10,my_r4=30;

int ancho_campo_texto_4=150;

int x_r5=45,y_r5=450,dx_r5=170,dy_r5=270;

int mx_r5=10,my_r5=30;

int x_r6=120,y_r6=280,dx_r6=210,dy_r6=85;

int mx_r6=10,my_r6=30;

int lf = 10;

int BAUD_RATE=9600;

String inString=null;

void setup () {

cp5 = new ControlP5(this);

cp5.addButton(“cargar_parametros_archivo”)

.setValue(0)

.setPosition(x_r4+40*mx_r4,y_r4+4*my_r4)

.setSize(ancho_boton+ancho_mitad_boton,alto_boton)

;

cp5.addTextfield(“nom_archivo”)

.setPosition(x_r2+2*mx_r2+ancho_campo_texto, y_r2+my_r2/3)

.setSize(150,25)

.setCaptionLabel(““)

.setColorBackground(color(84,153,199))

.setColorActive(color(100,0,0))

.setColorForeground(color(100,0,0))

.setFont(createFont(“times new roman”,20))

;

cp5.addTextfield(“para_nom_archivo”)

.setPosition(x_r4+25*mx_r4+ancho_campo_texto, y_r4+4*my_r4/3)

.setSize(150,25)

.setCaptionLabel(““)

.setColorBackground(color(84,153,199))

.setColorActive(color(100,0,0))

.setColorForeground(color(100,0,0))

.setFont(createFont(“times new roman”,20))

;

cp5.addTextfield(“tiempo_1”)

.setPosition(x_r4+mx_r4+ancho_campo_texto_4, y_r4+my_r4+dy_t/3)

.setSize(100,25)

.setCaptionLabel(““)

.setColorBackground(color(84,153,199))

.setColorActive(color(100,0,0))

.setColorForeground(color(100,0,0))

.setFont(createFont(“times new roman”,20))

;

cp5.addTextfield(“tiempo_2”)

.setPosition(x_r4+mx_r4+ancho_campo_texto_4, y_r4+my_r4+4*dy_t/3)

.setSize(100,25)

.setCaptionLabel(““)

.setColorBackground(color(84,153,199))

.setColorActive(color(100,0,0))

.setColorForeground(color(100,0,0))

.setFont(createFont(“times new roman”,20))

;

cp5.addTextfield(“tiempo_3”)

.setPosition(x_r4+mx_r4+ancho_campo_texto_4, y_r4+my_r4+7*dy_t/3)

.setSize(100,25)

.setCaptionLabel(““)

.setColorBackground(color(84,153,199))

.setColorActive(color(100,0,0))

.setColorForeground(color(100,0,0))

.setFont(createFont(“times new roman”,20))

;

cp5.addTextfield(“tiempo_4”)

.setPosition(x_r4+mx_r4+ancho_campo_texto_4, y_r4+my_r4+10*dy_t/3)

.setSize(100,25)

.setCaptionLabel(““)

.setColorBackground(color(84,153,199))

.setColorActive(color(100,0,0))

.setColorForeground(color(100,0,0))

.setFont(createFont(“times new roman”,20))

;

cp5.addTextfield(“tiempo_5”)

.setPosition(x_r4+mx_r4+ancho_campo_texto_4, y_r4+my_r4+13*dy_t/3)

.setSize(100,25)

.setCaptionLabel(““)

.setColorBackground(color(84,153,199))

.setColorActive(color(100,0,0))

.setColorForeground(color(100,0,0))

.setFont(createFont(“times new roman”,20))

;

cp5.addTextfield(“I_c”)

.setPosition(x_r4+mx_r4+ancho_campo_texto_4, y_r4+my_r4+16*dy_t/3)

.setSize(100,25)

.setCaptionLabel(““)

.setColorBackground(color(84,153,199))

.setColorActive(color(100,0,0))

.setColorForeground(color(100,0,0))

.setFont(createFont(“times new roman”,20))

;

cp5.addTextfield(“V_c”)

.setPosition(x_r4+mx_r4+ancho_campo_texto_4, y_r4+my_r4+19*dy_t/3)

.setSize(100,25)

.setCaptionLabel(““)

.setColorBackground(color(84,153,199))

.setColorActive(color(100,0,0))

.setColorForeground(color(100,0,0))

.setFont(createFont(“times new roman”,20))

;

cp5.addTextfield(“tipo_CDI”)

.setPosition(x_r4+mx_r4+ancho_campo_texto_4, y_r4+my_r4+22*dy_t/3)

.setSize(200,25)

.setCaptionLabel(““)

.setColorBackground(color(84,153,199))

.setColorActive(color(100,0,0))

.setColorForeground(color(100,0,0))

.setFont(createFont(“times new roman”,20))

;

cp5.addToggle(“tension_max”)

.setPosition(x_r5+10*mx_r5,y_r5+3*my_r5/2)

.setSize(50,20)

.setCaptionLabel(“on off”)

.setColorLabel(color(21,67,96))

.setColorBackground(color(235,245,251))

.setColorActive(color(26,82,118))

.setValue(false)

.setMode(ControlP5.SWITCH)

;

cp5.addToggle(“carga_culombios”)

.setPosition(x_r5+10*mx_r5,y_r5+6*my_r5/2)

.setSize(50,20)

.setCaptionLabel(“on off”)

.setColorLabel(color(21,67,96))

.setColorBackground(color(235,245,251))

.setColorActive(color(26,82,118))

.setValue(false)

.setMode(ControlP5.SWITCH)

;

cp5.addToggle(“descarga_resistencia”)

.setPosition(x_r5+10*mx_r5,y_r5+9*my_r5/2)

.setSize(50,20)

.setCaptionLabel(“on off”)

.setColorLabel(color(21,67,96))

.setColorBackground(color(235,245,251))

.setColorActive(color(26,82,118))

.setValue(false)

.setMode(ControlP5.SWITCH)

;

cp5.addToggle(“carga_descarga”)

.setPosition(x_r5+10*mx_r5,y_r5+12*my_r5/2)

.setSize(50,20)

.setCaptionLabel(“on off”)

.setColorLabel(color(21,67,96))

.setColorBackground(color(235,245,251))

.setColorActive(color(26,82,118))

.setValue(false)

.setMode(ControlP5.SWITCH)

;

cp5.addToggle(“conductimetro”)

.setPosition(x_r5+10*mx_r5,y_r5+15*my_r5/2)

.setSize(50,20)

.setCaptionLabel(“on off”)

.setColorLabel(color(21,67,96))

.setColorBackground(color(235,245,251))

.setColorActive(color(26,82,118))

.setValue(false)

.setMode(ControlP5.SWITCH)

;

/***************************************************************************

Configuration window GUI

***************************************************************************/

size(1040,740);

stroke(227,10,10);

println(Serial.list());

myPort = new Serial(this, Serial.list()[0], 9600);

myPort.clear();

myPort.buffer(80);

// Throw out the first reading, in case we started reading

// in the middle of a string from the sender.

inString = myPort.readStringUntil(lf);

inString = null;

background(513,123,20);

bot = loadShape(“ULPGC.svg”);

shape(bot, 22, 22, 72, 52);

shape(bot, −100,−100);

color(26,82,118);

String s_1=“ULPGC-University of Las Palmas de Gran Canaria”;

String s_2=“Interface for MCDI and MCI characterization”;

textSize(22);

fill(248, 249, 249);

text(s_1, 100, 45);

text(s_2, 100, 70);

}

/*********************************************************************************

Test configuration

**********************************************************************************/

void tension_max(boolean theFlag) {

if(theFlag==true) {

fichero = createWriter(“Ensayo.”+nom_archivo);

fichero.print(“Test 1: “);

fichero.println(“Charge Vcmax y self-discharge”);

fichero.print(“CDI type: “);

fichero.println(tipo_CDI);

fichero.print(“Time 1: “);

fichero.println(tiempo_1);

fichero.print(“Time 2: “);

fichero.println(tiempo_2);

fichero.print(“Time 3: “);

fichero.println(tiempo_3);

fichero.print(“Ic: “);

fichero.println(I_c);

fichero.print(“Vcmax: “);

fichero.println(V_c);

fichero.flush();

fichero.close();

// Ensayo 1: con consigna Vcmax y auto-descarga

inicio = 1;

String Vc_arduino=str(convierte_Vcmax_cuentas(V_c));

String Ic_arduino=str(convierte_Icons_cuentas(I_c));

String tiempo_3_arduino=str(convierte_tiempo_horas(tiempo_3));

String tiempo_1_arduino=str(convierte_tiempo_minutos(tiempo_1));

String tiempo_2_arduino=str(convierte_tiempo_segundos(tiempo_2));

String i=““;

String t1=““;

String t2=““;

String t3=““;

String ic=““;

String vcmax=““;

i=str(inicio);

t1=tiempo_1_arduino;

t2=tiempo_2_arduino;

t3=tiempo_3_arduino;

ic=Ic_arduino;

vcmax=Vc_arduino;

tipo_ensayo=“1”; // Ensayo a tensión máxima Vcmax

myPort.write(“c”);

myPort.write(i);

myPort.write(“,”);

myPort.write(t1);

myPort.write(“,”);

myPort.write(t2);

myPort.write(“,”);

myPort.write(t3);

myPort.write(“,”);

myPort.write(ic);

myPort.write(“,”);

myPort.write(vcmax);

hora_inicio=“Comienzo: “+hour()+”:”+minute()+”:”+second();

if(archivo==null){

}else{

archivo.flush();

archivo.close();

}

archivo = createWriter(nom_archivo);// Se crea un TXT

nom_archivo=“I-”+nom_archivo;

}else{

//STOP de CDI

String i=““+0;

String t1=““+0;

String t2=““+0;

String t3=““+0;

String ic=““+4095;

String vcmax=““+1000;

tipo_ensayo=“0”;

myPort.write(“c”);

myPort.write(i);

myPort.write(“,”);

myPort.write(t1);

myPort.write(“,”);

myPort.write(t2);

myPort.write(“,”);

myPort.write(t3);

myPort.write(“,”);

myPort.write(ic);

myPort.write(“,”);

myPort.write(vcmax);

if(archivo==null){

} else {

archivo.flush();

archivo.close();

}

}

}

void carga_culombios(boolean theFlag) {

if(theFlag==true) {

fichero = createWriter(“parameters.”+nom_archivo);

fichero.print(“Test 2: “);

fichero.println(“Current density”);

fichero.print(“CDI type”);

fichero.println(tipo_CDI);

fichero.print(“Time 1: “);

fichero.println(tiempo_1);

fichero.print(“Time 2: “);

fichero.println(tiempo_2);

fichero.print(“Time 3: “);

fichero.println(tiempo_3);

fichero.print(“Ic: “);

fichero.println(I_c);

fichero.print(“Vcmax: “);

fichero.println(V_c);

fichero.flush();

fichero.close();

//Ensayo 2: Carga eléctrica Q

/*

I=Culombios/tiempo ---> Con t2 es otro ensayo para carga y descarga

*/

inicio = 2;

String Vc_arduino=str(convierte_Vcmax_cuentas(V_c));

String Ic_arduino=str(convierte_Icons_cuentas(I_c));

String tiempo_3_arduino=str(convierte_tiempo_horas(tiempo_3));

String tiempo_1_arduino=str(convierte_tiempo_minutos(tiempo_1));

String tiempo_2_arduino=str(convierte_tiempo_segundos(tiempo_2));

String i=““+inicio;

String t1=““+tiempo_1_arduino;

String t2=““+tiempo_2_arduino;

String t3=““+tiempo_3_arduino;

String ic=““+Ic_arduino;

String vcmax=““+Vc_arduino;

tipo_ensayo=“2”;

myPort.write(“c”);

myPort.write(i);

myPort.write(“,”);

myPort.write(t1);

myPort.write(“,”);

myPort.write(t2);

myPort.write(“,”);

myPort.write(t3);

myPort.write(“,”);

myPort.write(ic);

myPort.write(“,”);

myPort.write(vcmax);

hora_inicio=“Begin: “+hour()+”:”+minute()+”:”+second();

if(archivo==null){

} else {

archivo.flush();

archivo.close();

}

archivo = createWriter(nom_archivo);

nom_archivo=“I-”+nom_archivo;

} else {

String i=““+0;

String t1=““+0;

String t2=““+0;

String t3=““+0;

String ic=““+4095;

String vcmax=““+1000;

tipo_ensayo=“0”;

myPort.write(“c”);

myPort.write(i);

myPort.write(“,”);

myPort.write(t1);

myPort.write(“,”);

myPort.write(t2);

myPort.write(“,”);

myPort.write(t3);

myPort.write(“,”);

myPort.write(ic);

myPort.write(“,”);

myPort.write(vcmax);

if(archivo==null){

} else {

archivo.flush();

archivo.close();

}

}

}

void descarga_resistencia(boolean theFlag) {

if(theFlag==true) {

inicio = 3;

String i=““+inicio;

String t1=““+tiempo_1;

String t2=““+tiempo_2;

String t3=““+tiempo_3;

String ic=““+I_c;

String vcmax=““+V_c;

tipo_ensayo=“3”;

myPort.write(“c”);

myPort.write(i);

myPort.write(“,”);

myPort.write(t1);

myPort.write(“,”);

myPort.write(t2);

myPort.write(“,”);

myPort.write(t3);

myPort.write(“,”);

myPort.write(ic);

myPort.write(“,”);

myPort.write(vcmax);

if(archivo==null){

} else {

archivo.flush();

archivo.close();

}

} else {

String i=““+0;

String t1=““+0;

String t2=““+0;

String t3=““+0;

String ic=““+4095;

String vcmax=““+1000;

tipo_ensayo=“0”;//CDI Stop

myPort.write(“c”);

myPort.write(i);

myPort.write(“,”);

myPort.write(t1);

myPort.write(“,”);

myPort.write(t2);

myPort.write(“,”);

myPort.write(t3);

myPort.write(“,”);

myPort.write(ic);

myPort.write(“,”);

myPort.write(vcmax);

if(archivo==null){}

else{

archivo.flush();

archivo.close();

}

}

}

void carga_descarga (boolean theFlag) {

if(theFlag==true) {

fichero = createWriter(“test_4.”+nom_archivo);

fichero.print(“Test 4: “);

fichero.println(“Charge and discharge of the CDI”);

fichero.print(“CDI type: “);

fichero.println(tipo_CDI);

fichero.print(“Time 1: “);

fichero.println(tiempo_1);

fichero.print(“Time 2: “);

fichero.println(tiempo_2);

fichero.print(“Time 3: “);

fichero.println(tiempo_3);

fichero.print(“Time 4: “);

fichero.println(tiempo_4);

fichero.print(“Time 5: “);

fichero.println(tiempo_5);

fichero.print(“Ic: “);

fichero.println(I_c);

fichero.print(“Vcmax: “);

fichero.println(V_c);

fichero.flush();

fichero.close();

inicio = 4;

String Vc_arduino=str(convierte_Vcmax_cuentas(V_c));

String Ic_arduino=str(convierte_Icons_cuentas(I_c));

String tiempo_3_arduino=str(convierte_tiempo_horas(tiempo_3));

String tiempo_1_arduino=str(convierte_tiempo_minutos(tiempo_1));

String tiempo_2_arduino=str(convierte_tiempo_segundos(tiempo_2));

String tiempo_4_arduino=str(convierte_tiempo_segundos(tiempo_4));

String tiempo_5_arduino=str(convierte_tiempo_horas(tiempo_5));

String i=““;

String t1=““;

String t2=““;

String t3=““;

String t4=““;

String t5=““;

String ic=““;

String vcmax=““;

i=str(inicio);

t1=tiempo_1_arduino;

t2=tiempo_2_arduino;

t3=tiempo_3_arduino;

t4=tiempo_4_arduino;

t5=tiempo_5_arduino;

ic=Ic_arduino;

vcmax=Vc_arduino;

tipo_ensayo=“4”;

myPort.write(“c”);

myPort.write(i);

myPort.write(“,”);

myPort.write(t1);

myPort.write(“,”);

myPort.write(t2);

myPort.write(“,”);

myPort.write(t3);

myPort.write(“,”);

myPort.write(t4);

myPort.write(“,”);

myPort.write(t5);

myPort.write(“,”);

myPort.write(ic);

myPort.write(“,”);

myPort.write(vcmax);

hora_inicio=“Comienzo: “+hour()+”:”+minute()+”:”+second();

if(archivo==null){

}else{

archivo.flush();

archivo.close();

}

archivo = createWriter(nom_archivo);

nom_archivo=“I-”+nom_archivo;

}else{

//STOP CDI

String i=““+0;

String t1=““+0;

String t2=““+0;

String t3=““+0;

String t4=““+0;

String t5=““+0;

String ic=““+4095;

String vcmax=““+1000;

tipo_ensayo=“0”;

myPort.write(“c”);

myPort.write(i);

myPort.write(“,”);

myPort.write(t1);

myPort.write(“,”);

myPort.write(t2);

myPort.write(“,”);

myPort.write(t3);

myPort.write(“,”);

myPort.write(t4);

myPort.write(“,”);

myPort.write(t5);

myPort.write(“,”);

myPort.write(ic);

myPort.write(“,”);

myPort.write(vcmax);

if(archivo==null){

} else {

archivo.flush();

archivo.close();

}

}

}

void conductimetro(boolean theFlag) {

if(theFlag==true) {

fichero = createWriter(“Test.”+nom_archivo);

fichero.print(“Test 5: “);

fichero.println(“conductimeter CDI”);

fichero.print(“CDI type: “);

fichero.println(tipo_CDI);

fichero.print(“Time 1: “);

fichero.println(tiempo_1);

fichero.print(“Time 2: “);

fichero.println(tiempo_2);

fichero.print(“Time 3: “);

fichero.println(tiempo_3);

fichero.print(“Ic: “);

fichero.println(I_c);

fichero.print(“Vcmax: “);

fichero.println(V_c);

fichero.flush();

fichero.close();

inicio = 5;

String Vc_arduino=str(convierte_Vcmax_cuentas(V_c));

String Ic_arduino=str(convierte_Icons_cuentas(I_c));

String tiempo_3_arduino=str(convierte_tiempo_horas(tiempo_3));

String tiempo_1_arduino=str(convierte_tiempo_minutos(tiempo_1));

String tiempo_2_arduino=str(convierte_tiempo_segundos(tiempo_2));

String i=““;

String t1=““;

String t2=““;

String t3=““;

String ic=““;

String vcmax=““;

i=str(inicio);

t1=tiempo_1_arduino;

t2=tiempo_2_arduino;

t3=tiempo_3_arduino;

ic=Ic_arduino;

vcmax=Vc_arduino;

tipo_ensayo=“5”;

myPort.write(“c”);

myPort.write(i);

myPort.write(“,”);

myPort.write(t1);

myPort.write(“,”);

myPort.write(t2);

myPort.write(“,”);

myPort.write(t3);

myPort.write(“,”);

myPort.write(ic);

myPort.write(“,”);

myPort.write(vcmax);

hora_inicio=“Begin: “+hour()+”:”+minute()+”:”+second();

if(archivo==null){

}else{

archivo.flush();

archivo.close();

}

archivo = createWriter(nom_archivo);// Se crea un TXT

nom_archivo=“I-”+nom_archivo;

}else{

//STOP de CDI

String i=““+0;

String t1=““+0;

String t2=““+0;

String t3=““+0;

String ic=““+4095;

String vcmax=““+1000;

tipo_ensayo=“0”;

myPort.write(“c”);

myPort.write(i);

myPort.write(“,”);

myPort.write(t1);

myPort.write(“,”);

myPort.write(t2);

myPort.write(“,”);

myPort.write(t3);

myPort.write(“,”);

myPort.write(ic);

myPort.write(“,”);

myPort.write(vcmax);

if(archivo==null){

} else {

archivo.flush();

archivo.close();

}

}

}

/*********************************************************************************

Interface configuration

**********************************************************************************/

void draw () {

// Comand DRAW

fill(255, 243, 224);

rect(x_r1,y_r1,dx_r1,dy_r1);

color(250,0,0,205);

fill(0, 102, 153);

text(“ ### Measured data ###”, x_r1+mx_r1, y_r1+my_r1);

text(“> vc_measu= “+dato_1, x_r1+mx_r1, y_r1+my_r1+dy_t);

text(“> ic_measu= “+dato_2, x_r1+mx_r1, y_r1+my_r1+2*dy_t);

text(“> Time 1= “+tiempo_1+” minutes”, x_r1+mx_r1, y_r1+my_r1+3*dy_t);

text(“> Time 2= “+tiempo_2+” seconds”, x_r1+mx_r1, y_r1+my_r1+4*dy_t);

text(“> Time 3= “+tiempo_3+” hours”, x_r1+mx_r1, y_r1+my_r1+5*dy_t);

text(“> Time 4= “+tiempo_4+” secons”, x_r1+mx_r1, y_r1+my_r1+6*dy_t);

text(“> Time 5= “+tiempo_5+” hours”, x_r1+mx_r1, y_r1+my_r1+7*dy_t);

text(“> Icons_c = “+I_c+” A”, x_r1+mx_r1, y_r1+my_r1+8*dy_t);

text(“> Vcmax = “+V_c+” V”, x_r1+mx_r1, y_r1+my_r1+9*dy_t);

text(“> CDI_type= “+tipo_CDI+” prototype”, x_r1+mx_r1, y_r1+my_r1+10*dy_t);

fill(255, 243, 224);

rect(x_r2,y_r2,dx_r2,dy_r2);

color(250,0,0,205);

fill(0, 102, 153);

text(“File name:”,x_r2+mx_r2, y_r2+my_r2);

text(nom_archivo,x_r2+mx_r2, y_r2+my_r2+dy_t);

text(hora_inicio,x_r2+mx_r2, y_r2+my_r2+2*dy_t);

text(“Saving: “+hour()+”:”+minute()+”:”+second(),x_r2+mx_r2, y_r2+my_r2+3*dy_t);

fill(255, 255, 255);

strokeWeight(1);

color(250,0,0,255);

strokeWeight(2);

// Configuración de la interfaz del panel de parámetros de entradas

fill(255, 355, 205);

rect(x_r4,y_r4,dx_r4,dy_r4);

color(250,0,0,205);

fill(0, 102, 153);

text(“ **** Input parameters **** “, x_r4+mx_r4, y_r4+my_r4);

text(“Timer_1(min): “, x_r4+mx_r4, y_r4+my_r4+dy_t);

text(“Timer_2(seg): “, x_r4+mx_r4, y_r4+my_r4+2*dy_t);

text(“Timer_3(h): “, x_r4+mx_r4, y_r4+my_r4+3*dy_t);

text(“Timer_4(seg): “, x_r4+mx_r4, y_r4+my_r4+4*dy_t);

text(“Timer_5(h): “, x_r4+mx_r4, y_r4+my_r4+5*dy_t);

text(“Icons_c(A): “, x_r4+mx_r4, y_r4+my_r4+6*dy_t);

text(“Vcmax(V): “, x_r4+mx_r4, y_r4+my_r4+7*dy_t);

text(“CDI type: “, x_r4+mx_r4, y_r4+my_r4+8*dy_t);

text(“Name.file:”,x_r4+29*mx_r4, y_r4+2*my_r4);

text(“Test.”+para_nom_archivo,x_r4+29*mx_r4, y_r4+2*my_r4+dy_t);

fill(255, 355, 205);

rect(x_r5,y_r5,dx_r5,dy_r5);

color(250,0,0,205);

fill(0, 102, 153);

text(“Panel”, x_r5+6*mx_r5, y_r5+my_r5);

text(“Vcmax “, x_r5+2*mx_r5, y_r5+my_r5+1*dy_t);

text(“Q”, x_r5+4*mx_r5, y_r5+my_r5+5*dy_t/2);

text(“Rdesc”, x_r5+mx_r5, y_r5+my_r5+8*dy_t/2);

text(“Ch-dis”, x_r5+mx_r5, y_r5+my_r5+11*dy_t/2);

text(“σ”, x_r5+4*mx_r5, y_r5+my_r5+14*dy_t/2);

fill(255, 243, 224);

rect(x_r6,y_r6,dx_r6,dy_r6);

color(255,255,0,0);

fill(255, 0, 0);

ellipse(x_r6+2*mx_r4, y_r6+3.5*my_r6/2, 20, 20);

text(estado_CDI,x_r6+4*mx_r6,y_r6+2.5*my_r6/1.25);

text(“Estado CDI”, x_r6+5*mx_r6, y_r6+0.85*my_r6);

}

void serialEvent (Serial myPort) {

try{

inString = myPort.readStringUntil(‘\n’);

inString = trim(inString);

if (inString != null) {

String[[] lista = split(inString,”,”);

dato_1=lista[0];

dato_2=lista[1];

dato_3=lista[4];

estado_CDI=“ “;

switch(int(dato_3)) {

case 1:

estado_CDI=“Precarga”;

break;

case 2:

estado_CDI=“Cargando”;

break;

case 3:

estado_CDI=“Auto-descarga”;

break;

case 4:

estado_CDI=“Finalizado”;

break;

case 0:

estado_CDI=“Stop”;

break;

}

/*********************************************************************************

Save data to file txt

**********************************************************************************/

guardar_datos=int(dato_3);

if(guardar_datos>3){

guardar_datos=0;

archivo.close();

}

if(tipo_ensayo==“0”){

guardar_datos=0;

}

if(guardar_datos>0){

archivo.print(year( )+” “); // Year

archivo.print(month( )+” “); // Month

archivo.print(day( )+” “); // Day

archivo.print(hour( )+” “); // Hours

archivo.print(minute( )+” “); // minutes

archivo.print(second( )+” “); // Seconds

archivo.print(tipo_ensayo+” “);

archivo.print(dato_3+” “);

archivo.print(dato_1+” “);

archivo.println(dato_2+” “);

archivo.flush();

}

}

}

catch(RuntimeException e) {

println(e);

}

}

/*********************************************************************************

**********************************************************************************/

public void cargar_parametros_archivo(int theValue) {

println(“Botón del Evento: “+theValue);

String lines[] = loadStrings(“Ensayo.”+para_nom_archivo);

String list[] = split(lines[1], ‘:’);

tipo_CDI=list[1];

list = split(lines[2], ‘:’);

tiempo_1 =list[1];

list = split(lines[3], ‘:’);

tiempo_2 =list[1];

list = split(lines[4], ‘:’);

tiempo_3 =list[1];

list = split(lines[5], ‘:’);

I_c =list[1];

list = split(lines[6], ‘:’);

V_c =list[1];

para_nom_archivo=“nombre.txt”;

}

/*********************************************************************************

Subrutines

**********************************************************************************/

int convierte_tiempo_horas(String t_string) {

float tiempo_float=0;

int tiempo_int_cuentas=0;

tiempo_float=float(t_string);

tiempo_int_cuentas=round(3600*tiempo_float*muestras_segundo);

return tiempo_int_cuentas;

}

int convierte_tiempo_minutos(String t_string) {

float tiempo_float=0;

int tiempo_int_cuentas=0;

tiempo_float=float(t_string);

tiempo_int_cuentas=round(60*tiempo_float*muestras_segundo);

return tiempo_int_cuentas;

}

int convierte_tiempo_segundos(String t_string) {

float tiempo_float=0;

int tiempo_int_cuentas=0;

tiempo_float=float(t_string);

tiempo_int_cuentas=round(tiempo_float*muestras_segundo);

return tiempo_int_cuentas;

}

int convierte_Icons_cuentas(String Icons_string) {

float Ic_float=0;

int Icons_int_cuentas=2048;

Ic_float=float(Icons_string);

Ic_float=Ic_float-.0;

Ic_float=−14741.88*Ic_float+2047.5;

Icons_int_cuentas=round(Ic_float);

return Icons_int_cuentas;

}

int convierte_Vcmax_cuentas(String Vcmax_string) {

float Vcmax_float=1.2;

int Vcmax_int_cuentas=0;

float sensibilidad=0.125;

float Voffset=0.7;

Vcmax_float=float(Vcmax_string);

Vcmax_float=(Vcmax_float+Voffset)*1000/sensibilidad;

Vcmax_int_cuentas=round(Vcmax_float);

return Vcmax_int_cuentas;

}
